# Supplementary material for: Identification of photoperiod-induced specific miRNAs in the adrenal glands of Sunite sheep (Ovis aries)
Source: Front Vet Sci. 2022 Jul 22;9:888207. doi: 10.3389/fvets.2022.888207 (PMC9354845; doi:10.3389/fvets.2022.888207)
Supplement: Supplementary file 1 [file Data_Sheet_1.ZIP › Supplementary file/miRDeep2 file.zip!/miRDeep2 file/Novel_Structure/SP42A3/Novel_552.pdf]

Provisional ID : NC_019463.2_12265
Score total : 18.7
Score for star read(s) : 3.9
Score for read counts : 12.4
Score for mfe : 0.9
Score for randfold : 1.6
Score for cons. seed :
Total read count : 36
Mature read count : 34
Loop read count : 0
Star read count : 2

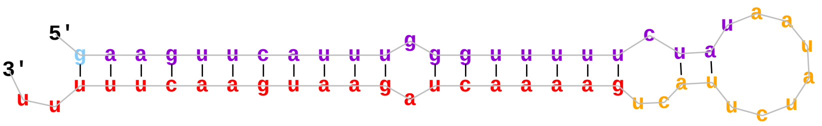

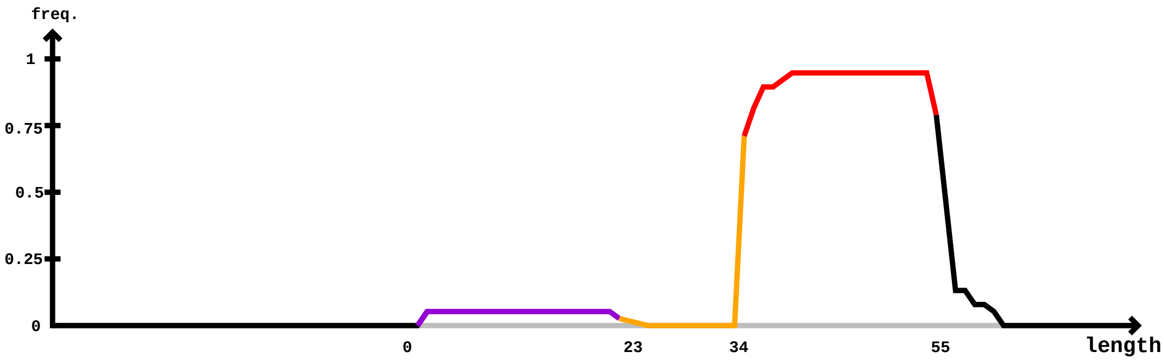


```
                                    Star                          Mature
5'- ucagauguucacuuagcauaaauauugguugcccaagaaguucauuuggguuuuucuauaauaucuuacugaaaacuagaaugaacuuuuuggccaacuuaauauaaccua  -3'   obs
    ucagauguucacuuagcauaaauauugguugcccaagaaguucauuuggguuuuucuauaauaucuuacugaaaacuagaaugaacuuuuuggccaacuuaauauaaccua         exp
    ....(((((.....)))))..((((((((((.(((((((((((((((.(((((((.((........))..))))))).))))))))))))))).)))).))))))......  reads   mm   sample
    .....................................aaguucauCuggguuuuucu......................................................  1       1    seq
    .....................................aaguucauuuggguuuuucuGu....................................................  1       1    seq
    ......................................................................Aaaaacuagaaugaacuuuu.....................  6       1    seq
    ......................................................................Aaaaacuagaaugaacuuuuu....................  17      1    seq
    ......................................................................gaaaacuagaaugaacuuuuu....................  1       0    seq
    ......................................................................Aaaaacuagaaugaacuuuuug...................  3       1    seq
    .......................................................................aaaacCagaaugaacuuuuu....................  3       1    seq
    .......................................................................aaaacuagaaugaacuuuuuggU.................  1       1    seq
    ........................................................................aaacCagaaugaacuuuuug...................  1       1    seq
    ........................................................................aaacuGgaaugaacuuuuuggc.................  1       1    seq
    ........................................................................aaacuUgaaugaacuuuuuggcca...............  1       1    seq
    ..........................................................................acuagaaugaacuuuuuggccaa..............  1       0    seq
    ...........................................................................cuCgaaugaacuuuuuggccaa..............  1       1    seq
```
